# Spectroscopic and deep learning-based approaches to identify and quantify cerebral microhemorrhages

**DOI:** 10.1038/s41598-021-88236-1

**Published:** 2021-05-21

**Authors:** Christian Crouzet, Gwangjin Jeong, Rachel H. Chae, Krystal T. LoPresti, Cody E. Dunn, Danny F. Xie, Chiagoziem Agu, Chuo Fang, Ane C. F. Nunes, Wei Ling Lau, Sehwan Kim, David H. Cribbs, Mark Fisher, Bernard Choi

**Affiliations:** 1 Beckman Laser Institute and Medical Clinic, University of California-Irvine, Irvine, CA USA; 2 Department of Biomedical Engineering, University of California-Irvine, Irvine, CA USA; 3 Department of Biomedical Engineering, Beckman Laser Institute Korea, Dankook University, Cheonan, 31116 Republic of Korea; 4 Massachusetts Institute of Technology, Cambridge, MA USA; 5 Albany State University, Albany, GA USA; 6 Department of Medicine, Division of Nephrology, University of California-Irvine, Irvine, CA USA; 7 Institute for Memory Impairments and Neurological Disorders, University of California-Irvine, Irvine, CA USA; 8 Neurology and Pathology and Laboratory Medicine, University of California-Irvine, Irvine, CA USA; 9 Department of Surgery, University of California-Irvine, Irvine, CA USA; 10 Edwards Lifesciences Center for Advanced Cardiovascular Technology, University of California-Irvine, Irvin, CA USA

**Keywords:** Neurology, Optical techniques

## Abstract

Cerebral microhemorrhages (CMHs) are associated with cerebrovascular disease, cognitive impairment, and normal aging. One method to study CMHs is to analyze histological sections (5–40 μm) stained with Prussian blue. Currently, users manually and subjectively identify and quantify Prussian blue-stained regions of interest, which is prone to inter-individual variability and can lead to significant delays in data analysis. To improve this labor-intensive process, we developed and compared three digital pathology approaches to identify and quantify CMHs from Prussian blue-stained brain sections: (1) ratiometric analysis of RGB pixel values, (2) phasor analysis of RGB images, and (3) deep learning using a mask region-based convolutional neural network. We applied these approaches to a preclinical mouse model of inflammation-induced CMHs. One-hundred CMHs were imaged using a 20 × objective and RGB color camera. To determine the ground truth, four users independently annotated Prussian blue-labeled CMHs. The deep learning and ratiometric approaches performed better than the phasor analysis approach compared to the ground truth. The deep learning approach had the most precision of the three methods. The ratiometric approach has the most versatility and maintained accuracy, albeit with less precision. Our data suggest that implementing these methods to analyze CMH images can drastically increase the processing speed while maintaining precision and accuracy.

Cerebral microhemorrhages (CMHs), which are detected as cerebral microbleeds on gradient-echo T2*-weighted magnetic resonance imaging (MRI), are associated with cognitive impairment^1^ and an increased risk of hemorrhagic and ischemic stroke^2^. CMHs are present in 20% of individuals over 60 years old and 40% of individuals over 80 years old^3,4^. In addition to normal aging^5,6^, CMHs are increased in several disease states, including cerebral amyloid angiopathy^7,8^, chronic kidney disease^9^, hypertension^10,11^, and cerebral autosomal dominant arteriopathy with subcortical infarcts and leukoencephalopathy (CADASIL)^12^. Despite the large number of medical conditions associated with CMHs, there remains a fundamental gap in understanding their mechanistic formation. One method that facilitates the study of CMH formation is the histological analysis of fixed tissue sections. (5–40 μm) stained with Perl’s Prussian blue. Current methods that identify and quantify CMHs are typically subjective and require tedious microscopic inspection. They rely on manual selection of Prussian blue-stained regions or use manual thresholding^13^, which is time and labor intensive. Recent advances in digital pathology address these limitations by eliminating the need for laborious staining^14^ and automating the quantitative analysis of the samples^15,16^.

Here, we set out to develop and compare three digital pathology approaches to automatically identify and quantify CMHs from sections stained with Prussian blue: (1) ratiometric analysis of RGB pixel values, (2) phasor analysis of RGB images, and (3) deep learning. The ratiometric method applies a threshold to the ratio of the red and green pixel intensities to the blue intensity-squared. The phasor analysis method applies a mask to the RGB values in the phasor space^17,18^. The deep learning approach uses a mask region-based convolutional neural network (R-CNN) to segment the RGB images^19^. We found that each approach performs well at identifying CMHs and quantifying the CMH area. Furthermore, the deep learning and ratiometric approaches performed better than the phasor analysis approach. We found that the deep learning approach had the most precision, while the ratiometric approach had good accuracy and the most versatility, but less precision than the deep learning approach.

## Materials and methods

**Figure 1:**
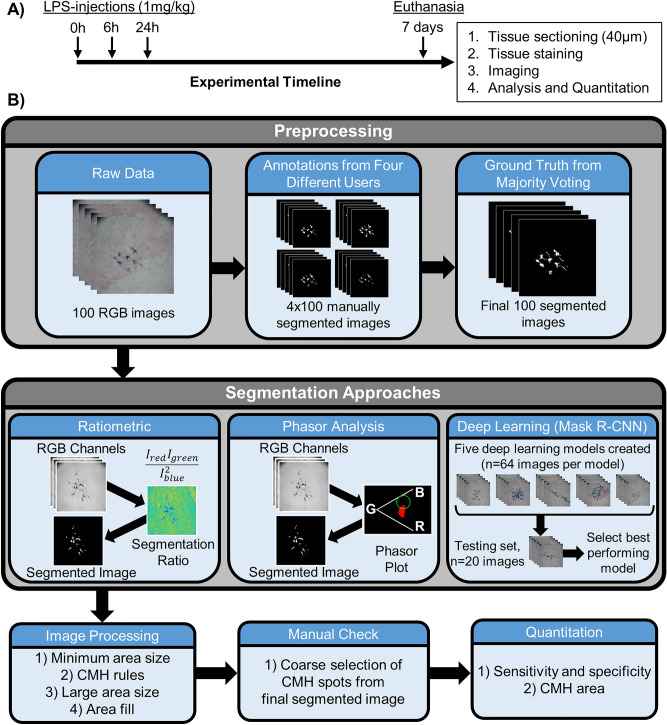
Overarching experimental timeline and data processing scheme. (**A**) Experimental timeline to induce cerebral microhemorrhages (CMHs), followed by tissue sectioning, staining, imaging, analysis, and quantitation. (**B**) Data preprocessing steps include: (1) acquiring raw color images of each CMH, (2) manually annotating color images by four users, and (3) determining the ground truth through majority voting. Three segmentation approaches (ratiometric, phasor analysis, and deep learning) were developed and compared. After the segmentation approaches, each set of results went through a series of image processing, data quality check, and quantitation steps (see the *Image Processing* section for details regarding each approach).

An overview of the experimental design, segmentation approaches, and processing steps are outlined in Fig. 1.

### Animal model

All animal procedures were executed in accordance with relevant guidelines and regulations approved by the University of California, Irvine’s Institutional Animal Care and Use Committee. Male C57BL/6 J mice (12 weeks old) were used (n = 4). Our study design complied with ARRIVE 2.0 guidelines. However, since our overall goal with the animal research was to generate a set of brain histology slides with exogenously labeled CMHs, we did not include a control group or incorporate blinding or randomization practices. To induce CMH formation^20^, we administered three doses of lipopolysaccharide (LPS) (1 mg/kg) to the mice at 0 h, 6 h, and 24 h via intraperitoneal injection (Fig. 1A). The mice fed and drank ad lib and received up to three daily doses of 1 mL saline subcutaneously. Mice were euthanized 7 days after the first injection with an overdose of sodium pentobarbital. After euthanasia, the chest cavity was immediately opened, and cardiac perfusion of saline followed by buffered formalin was performed. The brain was extracted and then immersed in buffered formalin for 24 h. The brain was then stored in PBS with 0.02% sodium azide^21^ until the time of sectioning.

### Sample preparation

Brains were sectioned into 40-µm coronal sections using a freezing microtome. Sections were stained with Prussian blue to detect hemosiderin, a marker of CMHs, using 10% hydrochloric acid and 5% potassium ferrocyanide trihydrate (Sigma, St. Louis, MO) for 30 min and rinsed in deionized water. Sections were then counterstained with Nuclear Fast Red (Sigma, St. Louis, MO) for 5 min, rinsed in deionized water, dehydrated, and mounted on a glass slide.

### Data acquisition and preprocessing

The stained sections were scanned for CMHs and imaged using a color camera (Chameleon3, FLIR, Wilsonville, OR) coupled to an inverted Nikon microscope. The light source from the Nikon microscope was used. One hundred unique images of Prussian blue-labeled CMHs were acquired and used for subsequent data analysis. Four independent users manually selected CMHs. Majority voting (i.e., at least two of the four users defined a pixel as a CMH) was done to determine the ground truth segmented data.

### Segmentation approaches

The key image processing steps for the segmentation approaches are shown in Supplementary Fig. 1.

### Ratiometric approach

The ratiometric approach was developed based on spectroscopic data acquired at 31 wavelengths (Supplementary Figs. 2 and 3). We defined a segmentation ratio using the red, green, and blue pixel intensities to spatially discriminate Prussian blue-labeled CMH pixels from background pixels: $segmentation ratio=\frac{{I}_{red}{I}_{green}}{{I}_{blue}^{2}}$. Segmentation ratio values that were below the specified threshold were included in the segmented image. We applied multiple segmentation ratio threshold values to (1) obtain segmented data for image processing, (2) calculate a receiver operator characteristic (ROC) curve^22^, and (3) determine the optimal segmentation ratio threshold. Please see the *Data Quantification and Statistical Analysis* section for how the optimal segmentation ratio was determined.

**Figure 2:**
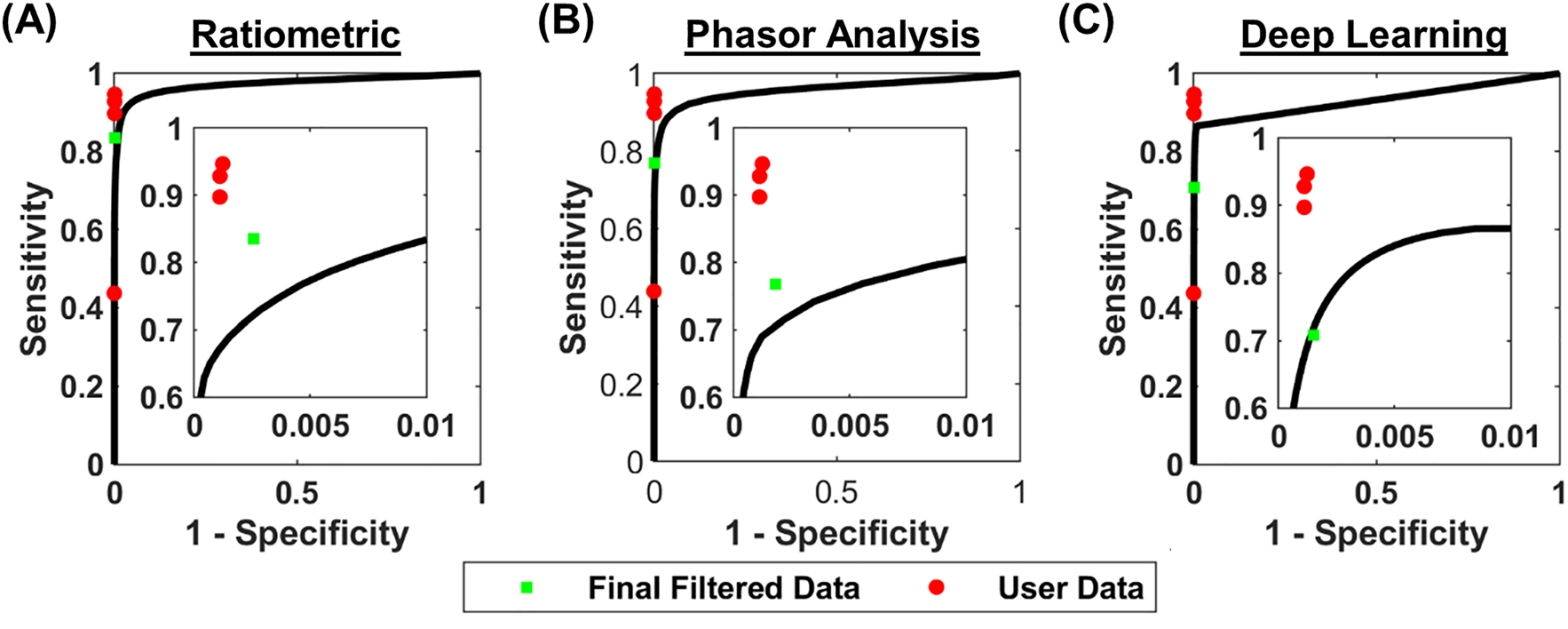
Receiver operating characteristics (ROC) curve for each approach to localize CMHs. (**A**) The ratiometric approach had an AUC of 0.973 and a final post-processed sensitivity and specificity of 0.835 and 0.997, respectively. (**B**) The phasor analysis approach had an AUC of 0.960 and a final post-processed sensitivity and specificity of 0.768 and 0.998, respectively. (**C**) The deep learning approach had an AUC of 0.932 and a final post-processed sensitivity and specificity were 0.708 and 0.998, respectively. The red circles represent the sensitivity and specificity for each independent user (relative to the ground truth), and the green squares represent the post-processed sensitivity and specificity for each approach.

**Figure 3:**
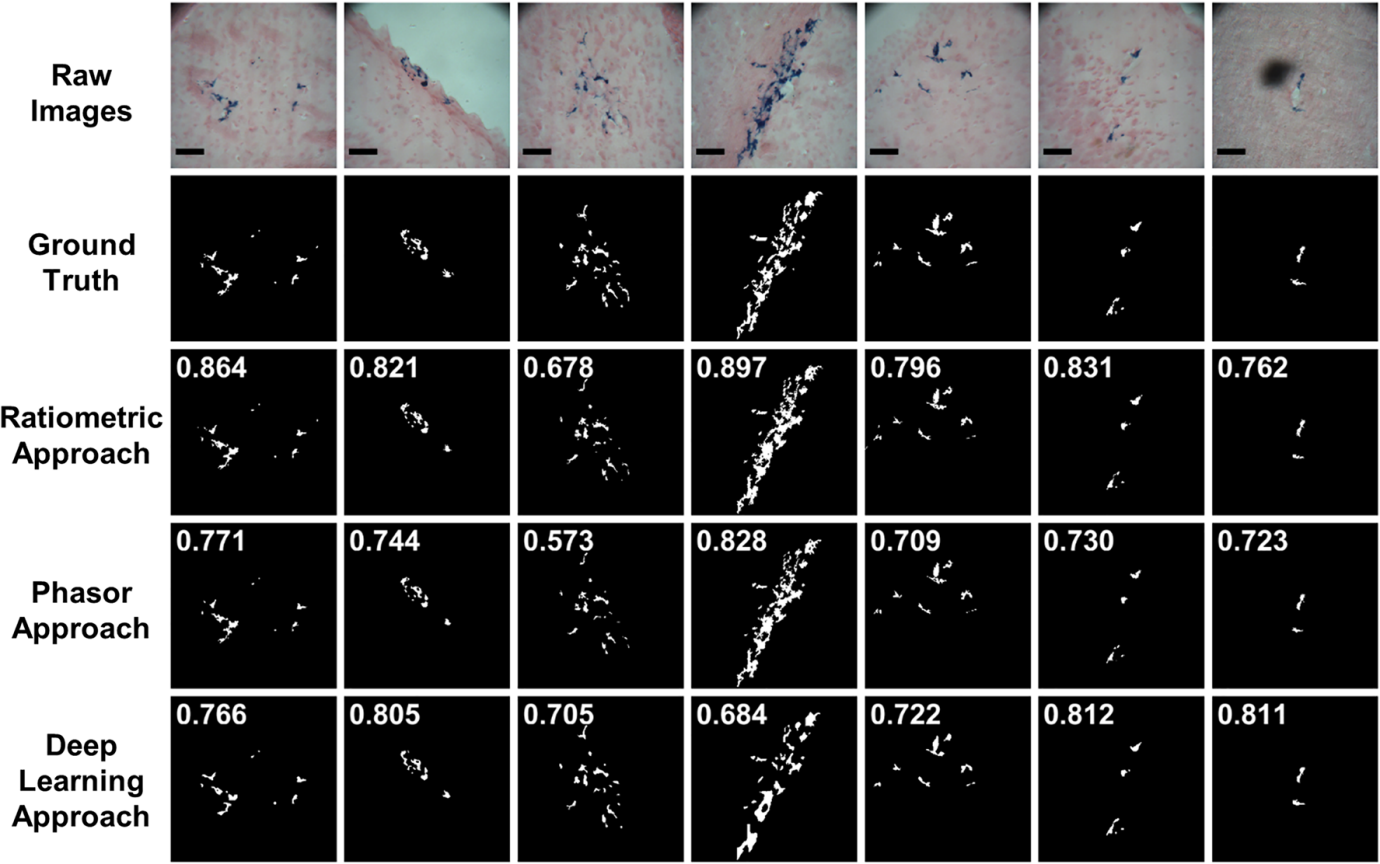
Representative examples (n = 7) of raw RGB images, ground truth data, and three segmentation approaches. The sensitivity is shown in the upper left of each image. The specificity of each image is greater than 0.990. The scale bar in the raw images is 50 µm.

### Phasor analysis approach

Phasor approaches in biomedical optics have been extensively used in fluorescence lifetime imaging microscopy (FLIM) to identify fluorophores in the collected images^23,24^. Recently, phasor approaches have been applied to spectroscopic data and RGB images from fluorescence microscopy and brightfield microscopy, respectively^17,18^. We applied a spectral phasor analysis technique to RGB images to isolate Prussian blue-labeled CMH pixels from background pixels. A discrete Fourier transform was performed at each pixel, which resulted in a phasor that consists of a real (H) and imaginary (S) component: $H=\sum_{l=1}^{3}{I}_{l}\mathrm{cos}\frac{2\pi l}{3}$ and $S=\sum_{l=1}^{3}{I}_{l}\mathrm{sin}\frac{2\pi l}{3}$ where $I$ is the collected intensity at channel $l$, and $l$ is the red, green, or blue channel. For each image, a plot of H vs. S was plotted to create a 2D phasor space.

To remap Prussian blue-labeled pixels from the phasor space plot to the original image, we found the bluest location in phasor space by minimizing x/y, where x is the row index and y is the column index of the phasor plot matrix. The center of a circular mask (green circle in Fig. 1B) was created from this point, and pixels enclosed by the circular mask in phasor space were remapped to a segmented image. We varied the size of the circular masks to (1) obtain segmented data for image processing, (2) calculate a ROC curve, and (3) determine the optimal mask in phasor space. Please see the *Data Quantification and Statistical Analysis* section for how the optimal circular mask was determined.

### Deep learning approach

For data preprocessing, the 100 CMH images were randomly divided into a training (n = 80) and testing (n = 20) set. The original 1000 × 1000 image resolution of the training set was downsampled to a resolution of 512 × 512 to reduce memory requirements and increase image processing speed. We applied a run-coding scheme to the 100 acquired CMH images^25^. To avoid overfitting, we augmented the training set by implementing random flip, rotations (90 ~ 270 degree), pixel multiplication with random values (0.8–1.5), and image blurring with Gaussian kernels (random sigma: 0 ~ 5) methods.

To identify and segment CMHs, we adopted a mask region-based convolutional neural network (R-CNN) deep learning algorithm developed by Matterport Inc.^26^. The Resnet-101 feature pyramid network model was employed as a backbone for the Mask R-CNN. The model was initialized using the weights obtained from the pre-training of the Microsoft common objects in context (MS-COCO)^27^ datasets through a transfer learning scheme^28^. Next, the network heads were trained for 20 epochs with a learning rate of 0.005. Then, all networks were trained for 60 epochs with a learning rate of 0.001. Finally, callback was set to stop training when the validation loss did not improve for ten epochs.

To assess consistency across our relatively small data set, we performed a fivefold cross validation. We randomly divided the 80 training set images into five groups (n = 16/group). Five models were created (n = 64 images/model) by leaving one group out per model. Each model was validated on the testing set. The Dice similarity coefficient^29^ was calculated using the testing, training, and combined data sets. Across the five different models, the Dice similarity coefficient ranged from 0.772 to 0.810 using the combined data sets, which shows consistency across the developed models. The model with the largest Dice similarity coefficient across the combined data set was used as the final deep learning model for further analysis. A ROC curve was calculated using the optimal deep learning model by applying different threshold values to the probability of each pixel being a CMH.

### Image processing

We empirically designed and performed the following image processing steps for all segmented images (ground truth, ratiometric, phasor, and deep learning). First, all segmented areas less than 5 µm^2^ were removed. Next, segmented areas less than 50 µm^2^ that were greater than 50 µm from the closest segmented spot were removed. For the ratiometric and phasor approaches, segmented areas larger than 6000 µm^2^ were removed. The phasor approach filled holes with an area of less than 1.73 µm^2^, which was empirically chosen. Finally, for the ratiometric and phasor approaches, a manual check was performed by drawing a coarse circle around all Prussian blue spots to remove pixels that were not CMHs. The deep learning approach did not require this step due to extremely high specificity and underestimation of CMH area.

### Data quantification and statistical analysis

ROC curves were calculated after the first two image processing steps. To obtain the final segmented image for each segmentation approach and subsequent data quantification, we performed one of the following steps:

1. Ratiometric approach: following all image processing steps, we found the median segmentation ratio threshold across all RGB images that resulted in the minimum percent error between the ground truth area and segmented area;
2. Phasor approach: after implementing all image processing steps, we found the mean radius of the circular mask in phasor space that, when remapped to the original image, resulted in the minimum percent error between the ground truth area and segmented area;
3. Deep learning approach: the final deep learning model had the highest Dice similarity coefficient across all images.

The final sensitivity and specificity were calculated for each approach using their final segmented image. To assess the agreement between the segmented areas and ground truth area, we calculated the intraclass correlation coefficient (ICC). We used a two-way random effects model with absolute agreement to calculate the ICC^30^. To further assess the agreement between the segmented areas and the ground truth, we generated Bland–Altman plots with 95% confidence limits of agreement^31^.

## Results

### Identifying CMH pixels

To assess the agreement between the regions of interest (ROIs) selected by the four independent users, we calculated their intraclass correlation coefficient (ICC) from the calculated CMH areas. The ICC was 0.954 (95% confidence interval 0.897–0.976), which demonstrates excellent agreement between the four independent users^30^. To assess the ability of each segmentation approach to identify CMH pixels, we compared the ground truth data to the segmented CMH data from each approach. Figure 2 shows ROC curves for the (A) ratiometric, (B) phasor analysis, and (C) deep learning approaches compared to the ground truth, and the final post-processed sensitivity and specificity. The ratiometric ROC curve has an AUC of 0.973, and a final post-processed sensitivity and specificity (green square) of 0.835 and 0.997, respectively (Fig. 2A). The phasor analysis ROC curve has an AUC of 0.960 and a final post-processed sensitivity and specificity (green square) of 0.768 and 0.998, respectively (Fig. 2B). The deep learning model ROC curve has an AUC of 0.932 and a final post-processed sensitivity and specificity (green square) of 0.708 and 0.998, respectively (Fig. 2C). Qualitative inspection shows good agreement among seven representative RGB images, ground truth data, and segmented images from each approach (Fig. 3). Interestingly, the final column of Fig. 3 shows a shadow artifact that is not detected as a CMH using any segmentation approach.

### Quantifying CMH area

**Figure 4:**
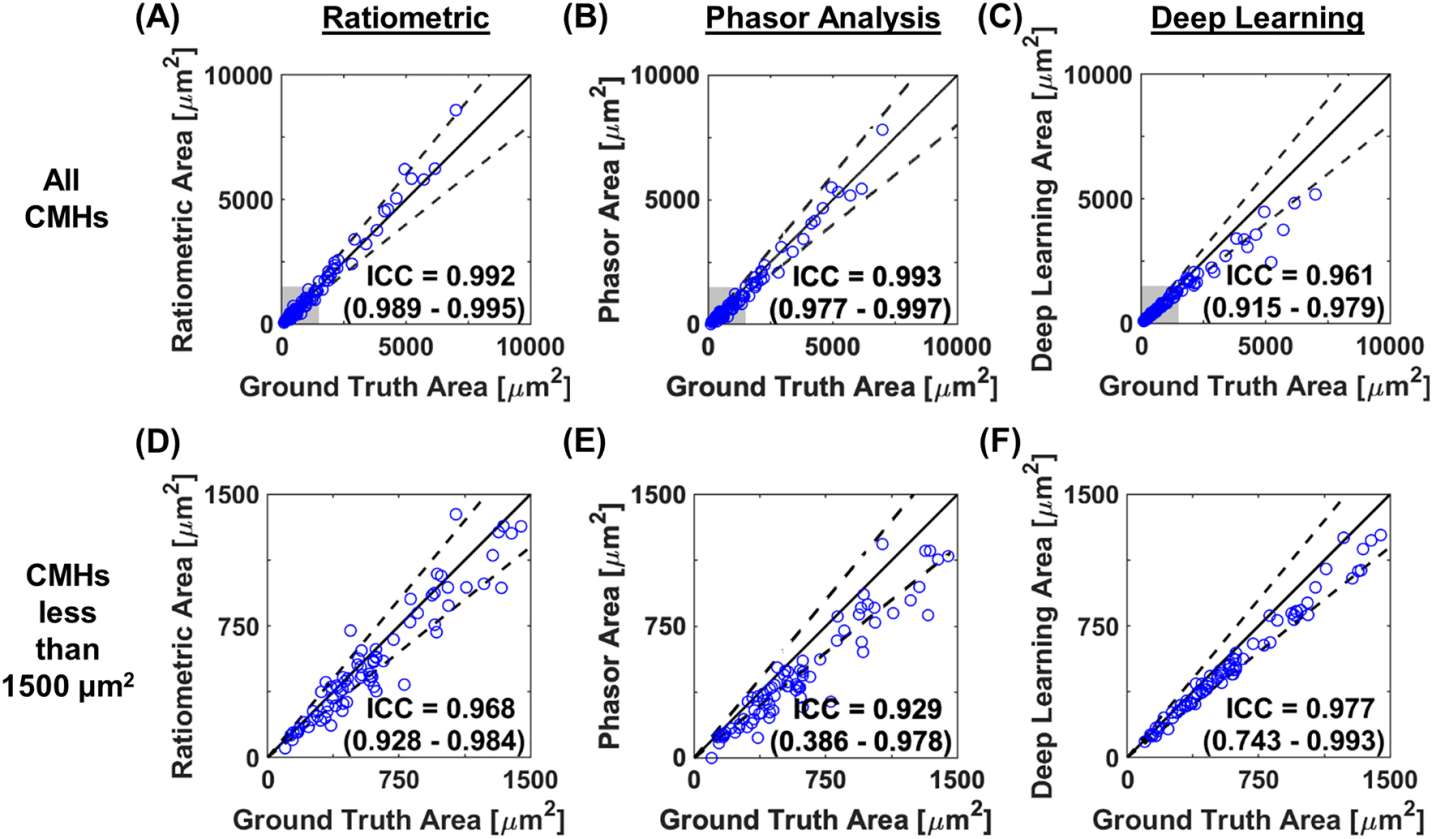
CMH area quantification between segmentation approaches and ground truth data with intraclass correlation coefficient (ICC). (**A**–**C**) Comparison between the ground truth and the segmentation approaches (**A**) ratiometric, (**B**) phasor analysis, and (**C**) deep learning for all calculated areas. (**D**, **E**) Magnified data of the shaded gray box shown in (**A**–**C**). Comparison between the ground truth and the segmentation approaches (**D**) ratiometric, (**E**) phasor analysis, and (**F**) deep learning for areas less than 1500 µm^2^. Intraclass correlation coefficients (ICC) and 95% confidence intervals are located in the lower right of each figure. In each graph (**A**–**F**), the solid black line is unity, and the dashed lines are ± 20% error.

To assess the agreement of the CMH area between each segmentation approach and the ground truth, we calculated the ICC. We examined the ICC using all CMH areas (n = 100), and CMH areas less than 1500 µm^2^ (n = 75). Using all calculated areas, the ICCs from the ratiometric, phasor, and deep learning approaches were 0.992 (95% confidence interval 0.989–0.995), 0.993 (95% confidence interval 0.977–0.997), and 0.961 (95% confidence interval 0.915–0.979), respectively (Fig. 4A–C). Using areas less than 1500 µm^2^, the ICCs from the ratiometric, phasor, and deep learning approaches were 0.968 (95% confidence interval 0.928–0.984), 0.929 (95% confidence interval 0.386–0.978), 0.977 (95% confidence interval 0.743–0.993), respectively (Fig. 4D–F). These results demonstrate excellent agreement between each developed approach and the ground truth (majority voting data). However, the ratiometric approach had the most consistent results compared to the phasor and deep learning approaches based on the 95% confidence interval.

**Figure 5:**
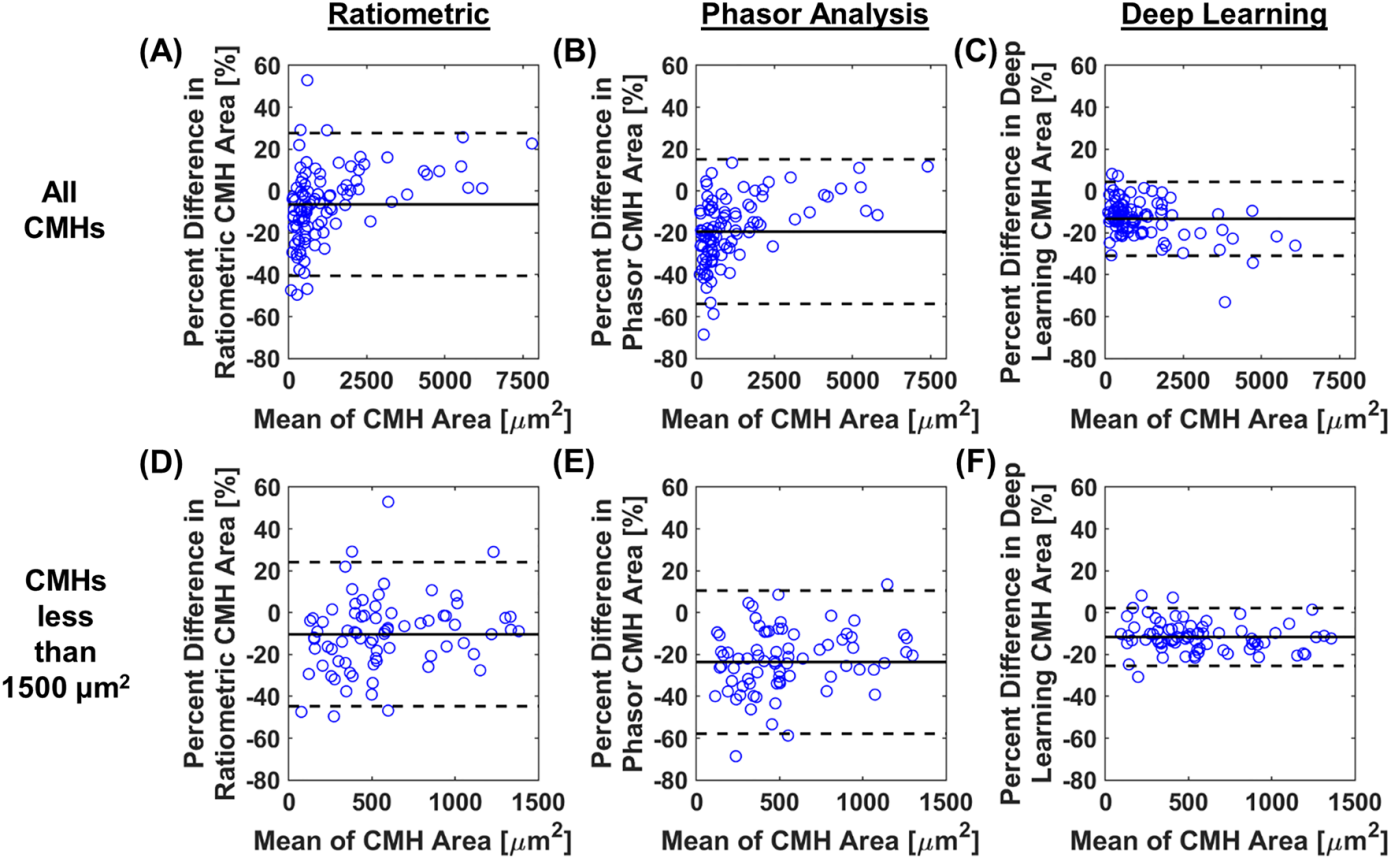
CMH area quantification between the segmentation approaches and ground truth data with the Bland–Altman plot. (**A**–**C**) Comparison between the ground truth and the segmentation approaches (**A**) ratiometric, (**B**) phasor analysis, and (**C**) deep learning for all calculated areas. (**D**–**E**) Comparison between the ground truth and the segmentation approaches (**D**) ratiometric, (**E**) phasor analysis, and (**F**) deep learning for areas less than 1500 µm^2^. In each graph (**A**–**F**), the y-axis is the percent difference between the areas from the selected segmentation approach (ratiometric, phasor, or deep learning) and the ground truth, and the x-axis is the mean area between the selected segmentation approach and ground truth. The solid black line is the mean difference and the dashed black lines are the 95% upper and lower limits of agreement.

To further assess the agreement between the segmented areas from each approach and the ground truth, we generated Bland–Altman plots. We examined the Bland–Altman plots using all CMH areas (n = 100), and CMH areas less than 1500 µm^2^ (n = 75). For each graph in Fig. 5, the y-axis is the percent difference between the areas from the selected segmentation approach (ratiometric, phasor, or deep learning) and the ground truth. The x-axis is the mean CMH area between the selected segmentation approach and ground truth. Each segmentation approach underestimates the calculated areas (negative percent difference), as is shown by the horizontal solid black line. This underestimation could be attributed to the users from the manual method not drawing on the exact border of the CMH, but around the CMH. The mean difference from the ratiometric approach (− 6.4% and − 10.3% for all CMH areas and less than 1500 µm^2^, respectively) was the closest to zero compared to the phasor (− 19.4% and − 23.6% for all CMH areas and less than 1500 µm^2^, respectively) and deep learning (− 13.3% and − 11.6% for all CMH areas and less than 1500 µm^2^, respectively) approaches. The proximity to zero suggests that the ratiometric approach may be most closely tied to the average CMH area. However, the deep learning approach had the smallest range for the upper and lower limits of agreement (35.2% and 27.6% for all CMH areas and less than 1500 µm^2^, respectively) compared to the ratiometric (68.1% and 68.8% for all CMH areas and less than 1500 µm^2^, respectively) and phasor (69.1% and 68.3% for all CMH areas and less than 1500 µm^2^, respectively) approaches. These results collectively suggest that the deep learning approach had the highest precision among the three developed approaches.

## Discussion

Histological sections remain the gold standard for assessing pathology from ex vivo tissue samples^14,32^. Clinical pathologists and researchers rely on data from these samples to guide patient management and gain mechanistic insights. However, tissue processing is laborious, requires extensive expertise, and is subjective^14^. Furthermore, gaining quantitative data from these samples is time exhaustive and subjective. Specifically, there are typically four steps required to characterize Prussian blue-stained CMHs. First, the brain is sectioned and stained with Prussian blue. Second, an individual visually inspects brain sections to identify and count CMHs with a brightfield microscope. Third, images of the identified CMHs are digitally acquired. Finally, a user manually outlines and adjusts RGB thresholds^13^ to quantify Prussian blue positivity or CMH area^20^. Each step is prone to error, variability, and subjectivity, and demands extensive personnel time.

In the present study, we set out to develop a digital processing approach to decrease the subjectivity and time involved in CMH quantitation. We developed and compared three approaches to identify and quantify CMHs stained with Prussian blue: (1) ratiometric, (2) phasor analysis, and (3) deep learning (Fig. 1). Our ratiometric approach used a combination of red, green, and blue pixel intensity values. Automated histological studies using different wavelengths of light have primarily been applied in cancer applications^33,34^. One breast cancer study used multispectral (16-wavelengths) and RGB data to spectrally unmix breast cancer markers^33^. The multispectral approach performed better than the RGB approach but is substantially more costly as it uses an expensive camera with an embedded liquid crystal tunable filter. A major advantage of our approach is that it can use the simple RGB values to study CMHs, which decreases the complexity and cost of the imaging system. Our second approach, the phasor analysis method, transforms the RGB image into phasor space through Fourier analysis. Phasor analysis in biomedical optics was first pioneered using FLIM data to identify fluorophores in images^23^. A proof-of-concept study applied spectral phasor analysis to brightfield, RGB data from a lymph node with metastatic cancer^18^. We leveraged off these advances in spectral phasor analysis of brightfield microscopy to separate Prussian blue-stained CMH pixels from background pixels. Although the phasor approach was the worst-performing approach, it may have advantages with new data sets from different microscopes, as it is based on global image analysis and requires limited or no reference information^17^. Our last approach, which uses deep learning, applies a mask region-based convolutional neural network (R-CNN). Many digital pathology approaches have used mask R-CNNs in applications of nuclei segmentation^12^ and cancer^35–37^. Similar to our study, these studies spatially identified specific features (e.g., nuclei). Our study is the first to apply a mask R-CNN to identify and quantify CMHs in histological images, where it performed well, similar to prior reports in other applications.

Our data demonstrate that each approach performs well at identifying CMHs and quantifying CMH areas (Figs. 2,3 and 4). A summary of the different approaches is shown in Table 1. The deep learning and ratiometric approaches performed better than the phasor analysis approach. The deep learning approach was the most precise method but had less versatility as it is not easily modifiable. This approach may require a lengthy re-training process when quantifying CMHs from different microscopes and image sets. The ratiometric approach had less precision than the deep learning approach. However, this approach accurately quantifies the CMH area and provides the user with more versatility to alter the threshold robustly. Therefore, depending on the needs of the user and the type of data set, the deep learning or ratiometric approach will be the optimal method to detect and quantify CMHs. Our data suggest that implementing these methods to analyze CMH data can drastically increase the processing speed while maintaining precision and accuracy.

**Table 1:** Comparison between manual method and three segmentation approaches for quantitation of cerebral microhemorrhages.

	Analysis process	Average time commitment per image	Modifiable	Sensitivity, specificity	Intraclass correlation coefficient (95% confidence interval)	Absolute area difference (µm^2^) (interquartile range)	Percent area difference (%) (interquartile range)
Manual approach	Subjective	10 min	N/A	N/A	N/A	N/A	N/A
Ratiometric approach	Semi-automated	15 s	Easy	0.835, 0.997	0.992 (0.989–0.995)	75.2 (33.3–172.3)	11.0 (4.7–21.2)
Phasor approach	Semi-automated	30 s	Moderate	0.768, 0.998	0.993 (0.977–0.997)	124.5 (55.2–255.2)	18.8 (9.3–29.4)
Deep learning approach	Automated	3 s	Difficult	0.708, 0.998	0.961 (0.915–0.979)	71.1 (33.6–167.7)	12.7 (8.8–18.9)

Our study does contain limitations that can be addressed with future work. Although the deep learning approach had the lowest sensitivity and ICC, the training data set was relatively small. Increasing the data set size could improve the deep learning results, especially for the larger sized CMHs, where the deep learning approach underestimated the CMH area. Second, the CMHs in our study were induced with LPS. Further testing of these approaches in other CMH models, such as chronic kidney disease^9^, hypertension^7^, and cerebral amyloid angiopathy^38–40^, in addition to human brain samples, are needed. Third, the algorithms have not been tested on different microscopes to assess if a calibration factor is needed to account for differences in camera spectral sensitivity and excitation spectrum. The deep learning approach may especially require a calibration or normalization step to ensure consistent input data into the deep learning model. Future work should test and assess the ability of the algorithms on different microscopes and with the use of multiple wavelengths of laser light on optically-cleared thick tissue sections to obtain three-dimensional (3D) information^41,42^. In addition, adding attention-based methods to the deep learning approach could help feature recognition and minimize background variability^43,44^. Fourth, the brain sections were visually scanned by users to find and acquire a digital image for each CMH. Using a slide scanner in conjunction with one of the developed approaches could drastically decrease the personnel time required to identify and quantify CMHs. One advantage that deep learning could provide that we did not investigate is to determine areas within images that contain CMHs and use the ratiometric or phasor approach for quantitation. Fifth, although Prussian blue is commonly used to detect CMHs, it does not detect newly formed CMHs, nor distinguish between hemoglobin-derived (hemosiderin) and nonhemoglobin-derived iron^13^. Combining our Prussian blue-developed approaches with future algorithms that use fresh CMHs stained with hematoxylin and eosin (H&E)^45^ may help detect non-acute and acute CMHs. Finally, in this work, we only focused on the analysis of CMH images labeled with exogenous Prussian blue. Future work that virtually stains samples through machine learning of autofluorescent and absorption features may minimize inter-batch variability due to staining, which could revolutionize the histological pipeline for examining CMHs by removing the manual tissue staining step^14,46,47^.

In conclusion, we developed and compared three segmentation approaches: (1) ratiometric, (2) phasor analysis, and (3) deep learning to identify and quantify CMHs stained with Prussian blue. The developed approaches can substantially reduce the considerable personnel and time commitment required to quantify CMHs in individual histological sections. The deep learning and ratiometric approaches performed better than the phasor analysis approach. We found that the deep learning approach had the most precision; however, the ratiometric approach had good accuracy and the most versatility, but less precision than the deep learning approach. Our data suggest that implementing these methods to analyze CMH images can drastically increase the processing speed, while maintaining precision and accuracy.

## Supplementary Information

Supplementary Information
