# Supplementary material for: Spectroscopic and deep learning-based approaches to identify and quantify cerebral microhemorrhages
Source: Sci Rep. 2021 May 21;11:10725. doi: 10.1038/s41598-021-88236-1 (PMC8140127; doi:10.1038/s41598-021-88236-1)
Supplement: Supplementary file 1 — Supplementary Information [file 41598_2021_88236_MOESM1_ESM.docx]

***Title***: Spectroscopic and deep learning-based approaches to identify and quantify cerebral microhemorrhages

Christian Crouzet^1^, Gwangjin Jeong^2^, Rachel H Chae^3^, Krystal T LoPresti^1,4^, Cody E Dunn^1,4^, Danny F Xie^1,4^, Chiagoziem Agu^1,5^, Chuo Fang^6^, Ane CF Nunes^7^, Wei Ling Lau^7^, Sehwan Kim^2^, David H Cribbs^8^, Mark Fisher^1,6^, and Bernard Choi^1,4,9,10^

Affiliations:

1 Beckman Laser Institute and Medical Clinic, University of California, Irvine, CA, USA

2 Department of Biomedical Engineering, Beckman Laser Institute Korea, Dankook University, Cheonan, 31116, Republic of Korea

3 Massachusetts Institute of Technology, Cambridge, MA, USA

4 Department of Biomedical Engineering, University of California, Irvine, CA, USA

5 Albany State University, Albany, Georgia, USA

6 Neurology and Pathology & Laboratory Medicine, University of California, Irvine, CA, USA

7 Medicine, Division of Nephrology, University of California, Irvine, CA, USA

8 Institute for Memory Impairments and Neurological Disorders, University of California, Irvine, CA, USA

9 Department of Surgery, University of California, Irvine, CA, USA

10 Edwards Lifesciences Center for Advanced Cardiovascular Technology, University of California, Irvine, CA, USA

**Corresponding author name and e-mail**: Bernard Choi, choib@uci.edu

***Supplemental Material:***

***Materials and Methods***

The image processing steps for each approach are shown in Supplementary Figure 1 and described in detail in the main body of the manuscript.


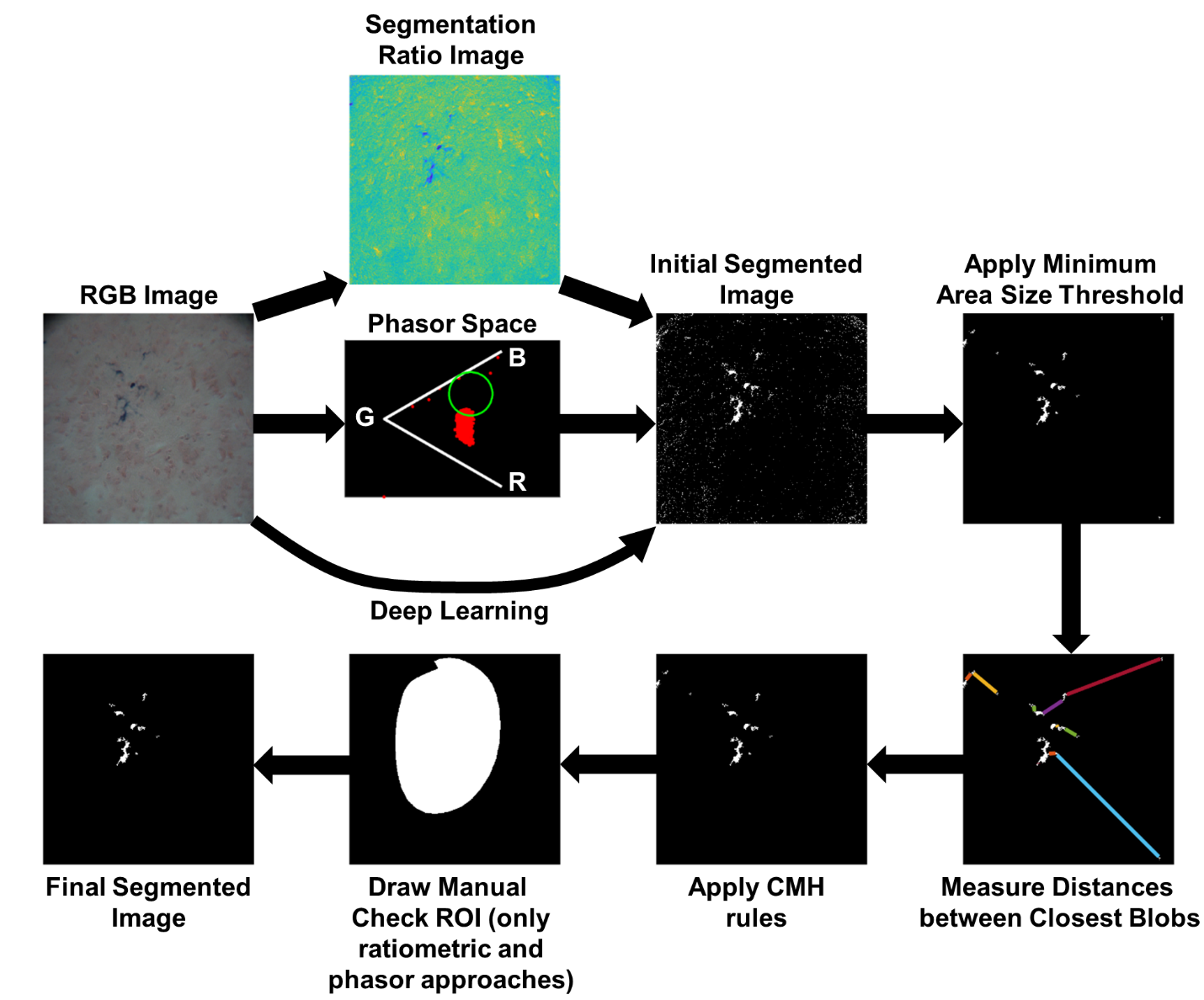


**Supplementary Figure 1: Image processing workflow.** An RGB image is converted into an initial segmented image for each algorithm in the following ways: 1) ratiometric: apply the segmentation ratio ($\frac{I_{red}I_{green}}{I_{blue}^{2}}$) to each pixel of the RGB image and apply a threshold to the ratio, 2) spectral phasor analysis: convert each pixel of the RGB image to the phasor space and apply a mask (green circle) to the phasor space, or 3) deep learning: apply the mask region-based convolutional neural network (R-CNN) black box processing. After the initial segmented image, a minimum size threshold is applied where areas smaller less than 5 µm^2^ were removed. Then, we measured the distance between each blob. We applied the CMH rule that segmented areas less than 50 µm^2^ that were greater than 50 µm from the closest segmented spot were removed. Finally, for the ratiometric and phasor approaches, a manual check was performed by drawing a coarse, enclosed circle around all Prussian blue spots to create a mask that removes pixels outside the mask to obtain the final segmented image. This final step was not needed for the deep learning approach since there were not stray CMH pixels like there were for the ratiometric and phasor approaches.

*Multispectral data acquisition and analysis*

To obtain multispectral data from the Prussian blue slides, we coupled a Nuance Multispectral Imaging System (PerkinElmer, Hopkinton, MA) to an inverted Nikon microscope. Data were acquired using the Nuance 3.0.2 software (PerkinElmer, Inc., Waltham, MA) from 420-720nm in 10nm steps. To keep intensity consistent from image to image, we used the auto exposure feature at 80% of the maximum intensity at each wavelength. Eighteen multispectral data sets were used for data analysis. Regions of interest (ROIs) were manually selected by outlining pixels that were positively-labeled by Prussian blue. A total of 182 Prussian blue ROIs and 144 background ROIs were selected. We used a two-tailed ranked-sum test to assess differences between the Prussian blue and background intensities at each wavelength.

***Results***

To qualitatively assess multispectral intensity changes, we examined the images at each of the acquired 31 wavelengths (Supplementary Figure 2). The data shows that the CMH is not visually apparent from 420nm to 530nm but is seen from 540nm to 720nm. Furthermore, the counterstain, Nuclear Fast Red, is visible from 490nm to 570nm. Quantitative comparison of background pixels and CMHs labeled with Prussian blue are shown in Supplementary Figure 3. There is no significant difference at blue wavelengths 440-470nm, but outside this range, the intensity values differ (p < 0.05), especially in the red wavelength range (600-720nm).


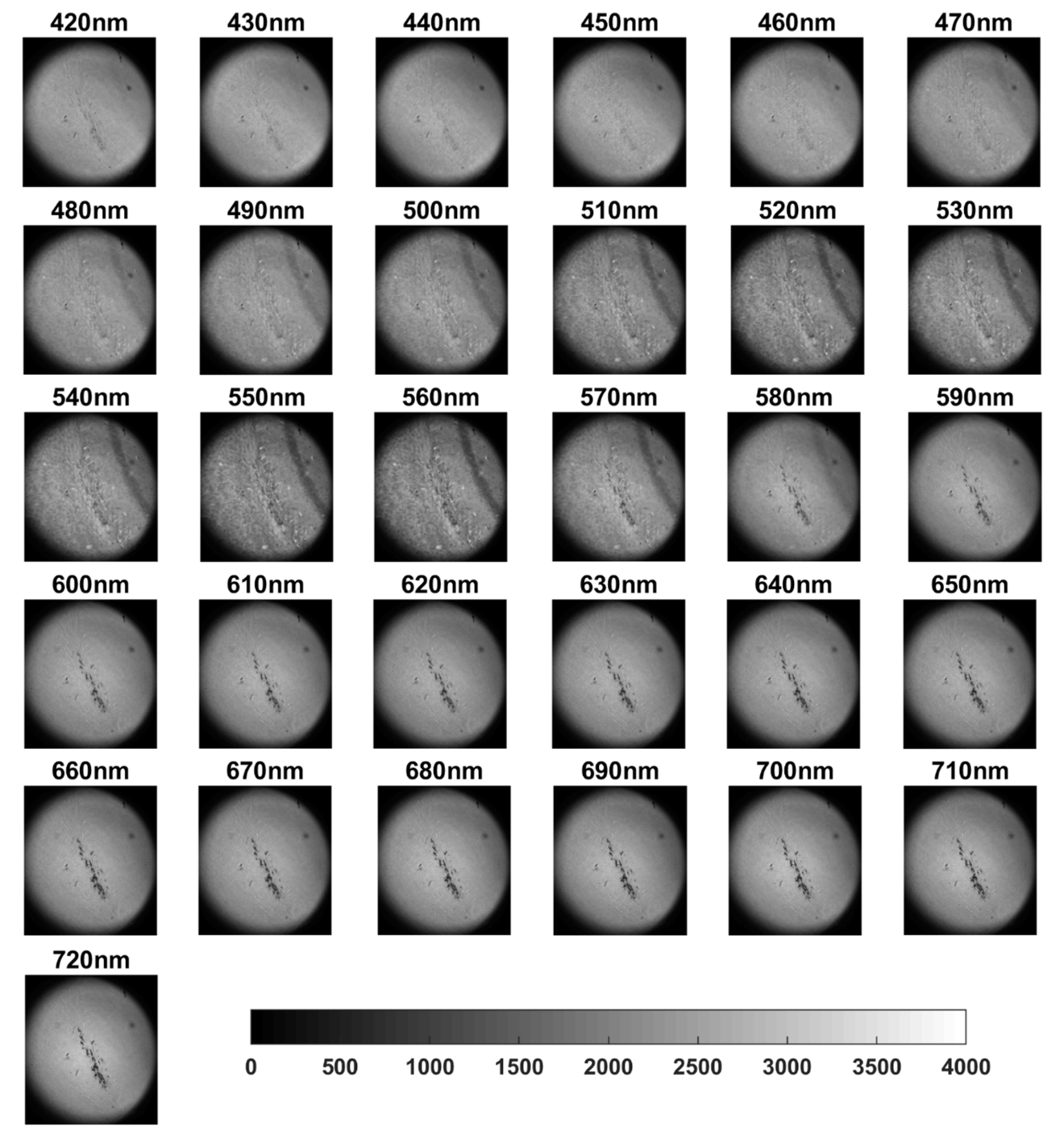


**Supplementary Figure 2: Qualitative assessment of CMH visibility with multispectral imaging.**


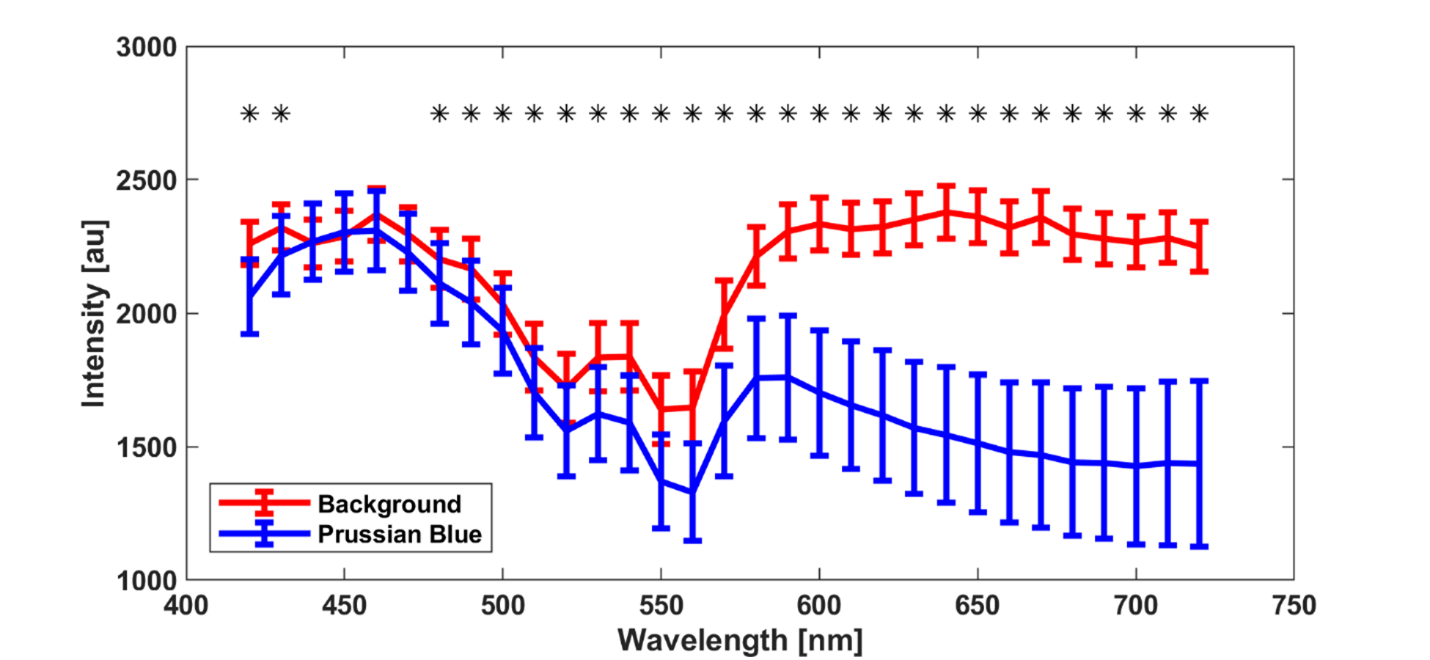


**Supplementary Figure 3: Quantitative comparison between CMH and background pixels with multispectral imaging.** Error bars are standard deviation. * represents p < 0.05
